# Self‐Administered Etripamil Nasal Spray Slows Ventricular Rate in Patients With Atrial Fibrillation: A Post Hoc Analysis of the NODE‐303 Study

**DOI:** 10.1111/jce.70287

**Published:** 2026-02-10

**Authors:** Paul Dorian, Marco Alings, Benoit Coutu, James E. Ip, Felipe A. Martinez, Jonathan P. Piccini, Bruce S. Stambler, Muhammad Sheikh, Silvia Shardonofsky, David B. Bharucha, A. John Camm

**Affiliations:** ^1^ Division of Cardiology, St. Michael's Hospital University of Toronto Toronto Ontario Canada; ^2^ Amphia Ziekenhuis Breda North Brabant Netherlands; ^3^ Department of Medicine, University of Montreal; Cardiac Electrophysiology Department Centre Hospitalier Universitaire de Montréal Montreal Québec Canada; ^4^ Division of Cardiology, Weill Cornell Medicine, New York Presbyterian Hospital New York New York USA; ^5^ Universidad Nacional de Córdoba; Instituto DAMIC/Fundacion Rusculleda Córdoba Argentina; ^6^ Department of Medicine Duke University School of Medicine Durham North Carolina USA; ^7^ Piedmont Heart Institute Atlanta Georgia USA; ^8^ Milestone Pharmaceuticals Charlotte North Carolina USA; ^9^ Milestone Pharmaceuticals Montreal Québec Canada; ^10^ St. George's University of London London England UK

**Keywords:** arrhythmia, atrial fibrillation with rapid ventricular rate, etripamil, NODE‐303, paroxysmal supraventricular tachycardia, self‐administered

## Abstract

**Introduction:**

Currently, there are no available fast‐acting agents to control the ventricular rate (VR) during atrial fibrillation (AF) episodes that can be self‐administered by patients on an as‐needed basis without medical supervision. Here, we aim to evaluate the effect of intranasal etripamil on VR in patients treating presumed paroxysmal supraventricular tachycardia (PSVT) episodes later identified as AF.

**Methods:**

NODE‐303 is a phase 3, multicenter, open‐label study to evaluate the safety and efficacy of self‐administered intranasal etripamil. The study enrolled adult (≥ 18 years) patients with prior documented PSVT. Patients self‐administered 70 mg of intranasal etripamil after applying an ECG monitor and performing a vagal maneuver. An optional second 70 mg dose was permitted if symptoms persisted after 10 min. This post hoc analysis specifically focused on AF patients (those with presumed PSVT episodes subsequently identified as AF) and evaluated the change from baseline VR (beats per minute, bpm), calculated using patient ECG data that was recorded through 60 min after drug administration, during an etripamil‐treated AF episode.

**Results:**

Of 1116 total enrolled patients, 503 self‐administered ≥ 1 dose of etripamil for presumed PSVT, and 18 had ≥ 1 episode(s) later identified as AF with adequate ECG data. AF episode baseline median VR (range) was 127 bpm (79, 164), and the average maximum reduction in VR was 27.4 bpm ± 6.1 at 22 min. VR reduction was sustained for at least 30 to 60 min (median VR reduction −22 and −18 bpm, respectively). No serious adverse events or adverse events of special interest were observed, including bradyarrhythmias, AV block, or sinus pauses ≥ 3 s, and treatment‐emergent adverse events were mild to moderate, transient, and related to the nasal administration route.

**Discussion:**

In this post hoc subgroup analysis of the open‐label NODE‐303 trial, self‐administered etripamil nasal spray led to a clinically significant and sustained VR reduction in patients with symptomatic AF. Further studies are needed to confirm efficacy in a broader AF population.

**Conclusion:**

Self‐administered etripamil may help to acutely control symptomatic AF‐RVR episodes outside of the healthcare setting.

**Clinical Trial Registration:**

NCT04072835.

## 1 Introduction

Atrial fibrillation (AF) is the most common sustained arrhythmia, affecting over 37 million people worldwide [1, 2, 3, 4]. Patients with AF often experience a rapid ventricular rate (AF‐RVR), frequently requiring acute care and/or emergency department visits [5]. Standard treatments for acute rate control include intravenous beta‐blockers, intravenous calcium channel (CCB) blockers, or electrical cardioversion [3, 6]. Orally administered AV nodal blockade may be used but have a slow onset relative to IV treatment and may cause bradycardia post‐conversion [3, 7]. Currently, there are no available rapidly acting agents to slow ventricular rates (VR) that can be self‐administered by patients outside of the healthcare setting.

Etripamil is a fast‐acting, non‐dihydropyridine L‐Type CCB, uniquely formulated for intranasal self‐administration outside of the healthcare setting. The drug prolongs AV‐nodal refractoriness and has a rapid onset of action (T_max_ ≤ 7 min) [8]. Etripamil, in development for paroxysmal supraventricular tachycardia (PSVT) and AF‐RVR, has shown efficacy and safety in rapidly terminating PSVT with reduced healthcare utilization [9, 10, 11]. In NODE‐303, patients self‐administered intranasal etripamil for presumed PSVT. This post hoc analysis evaluates the efficacy and safety of etripamil nasal spray in NODE‐303 patients, who were subsequently determined to have AF when electrocardiographic monitoring was reviewed [11].

## 2 Methods

### 2.1 Study Design

NODE‐303 (NCT04072835, clinicaltrials.gov) is a phase 3, multicenter, open‐label study to evaluate the safety and efficacy of self‐administered etripamil in adult patients with prior documented PSVT [12, 13]. Study methods and inclusion/exclusion criteria, which required a history of PSVT and allowed for a possible additional history of AF, have been described previously, with safety and efficacy results [14]. Patients with symptoms of congestive heart failure (II to IV), history of ACS during the last 6 months, history or evidence of pre‐excitation, or second‐third degree AV block, severe ventricular arrhythmia, severe symptoms of hypotension experienced during PSVT episodes or history of syncope were excluded from the NODE‐303 trial. Patients, upon experiencing PSVT symptoms, were instructed to first apply an electrocardiographic (ECG) monitor, and if symptoms persisted despite a vagal maneuver, to then self‐administer intranasal etripamil (70 mg). If symptoms persisted for 10 min (min), an optional second dose of etripamil 70 mg could be self‐administered. VR was captured by continuous ECG monitoring for ≥ 1 h after the onset of symptoms. All ECG recordings were evaluated by the study's medical staff and confirmed by expert review. Adverse events of special interest (AESI) occurring within 24 h after administration were tachyarrhythmias, bradyarrhythmias, AV block, pauses ≥ 3 s, hypotension, and syncope.

### 2.2 Post Hoc Analysis

We analyzed the continuous ECG data in patients with AF at the time of monitor application. AF‐RVR was defined as VR ≥ 110 bpm at the time of drug administration. For episodes, the start time of ECG recording was considered as the baseline time (*T* = 0 min) and, if drug administration was not demarcated by an event‐marker it was considered to have occurred at *T* = 0. VR values at the start of the ECG recording and at 1‐min intervals were calculated for each episode up to 60 min post‐drug self‐administration. VR was recorded as the number of heartbeats during a 1‐min window. Any VR data were excluded after conversion to normal sinus rhythm (NSR) during the monitoring period. Differences in VR from baseline were calculated at each time point and reported by descriptive statistics.

## 3 Results

### 3.1 Baseline Demographics

NODE‐303, enrolled 1116 patients; 503 (45.1% of those enrolled) patients self‐administered at ≥ 1 dose of etripamil; 26 episodes in 21 patients were adjudicated as AF episodes, including in 5 patients with 2 AF episodes each. In total, 21 episodes in 18 patients had quality data available for analysis and were included in the assessment (Table 1); of these, 17 episodes had AF‐RVR (VR ≥ 110 bpm at the time of drug administration) and 4 had AF without RVR (VR < 110 bpm at the time of drug administration).

**Table 1:** Baseline characteristics.

NODE‐303 Enrolled, *n* = 1116	
Patients with ≥ 1 etripamil‐treated episode, *n* (%)	503 (45.1)
Post Hoc Safety Analysis	*n* = 21
Adjudicated AF episodes	26
Post Hoc Efficacy Analysis	*n* = 18
Adjudicated AF episodes with ECG data	21
Adjudicated AF‐RVR^a^ episodes with ECG data	17
Characteristics of patients with adjudicated AF episode, *n* = 21	
Mean age, years	57.4
SD	15.6
Female patients, n (%)	11 (52.4)
Use of concomitant medications of interest^b^, *n* (%)	
β blockers	9 (48.3)
CCBs	3 (14.2)
β blockers or CCBs	10 (47.6)
β blockers and CCBs	1 (4.8)
None	10 (47.6)

Abbreviations: AF, atrial fibrillation; AF‐RVR, atrial fibrillation with rapid ventricular rate; CCB, calcium channel blocker; ECG, electrocardiograph; SD, standard deviation; SEM, standard error of mean; VR, ventricular rate.

^a^ AF‐RVR was defined as a VR of at least 110 bpm at the time of drug administration.

^b^ Concomitant medications refer to those acting on the atrioventricular node and used by a patient within 30 days prior to the screening visit. A patient could be taking more than one medication.

### 3.2 Effect on AF Episode VR and Safety of Self‐Administered Etripamil

**Figure 1:**
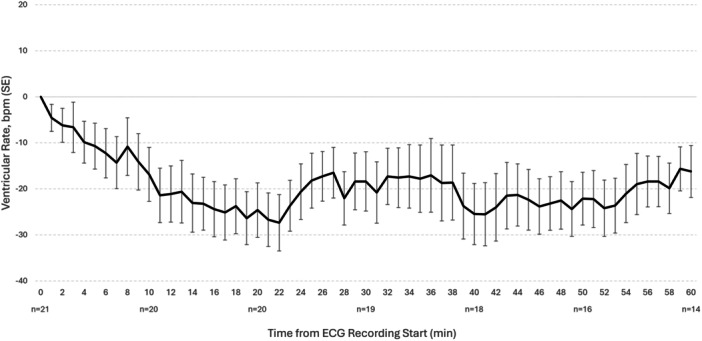
Effect of self‐administered etripamil on AF episode VR, post hoc efficacy population. VR (black, solid line) ± standard error recorded per minute after the start of ECG recording for 60 min. The start of ECG recording (time = 0) was used as an estimate for the time of etripamil administration. bpm, beats per minute; ECG, electrocardiograph; SE, standard error; VR, ventricular rate. There is some brief, unmeasured delay between drug administration and the time of start of the ECG recording, which may affect the latency to drug effects.

Baseline VR ± standard error of the mean (SEM) was 129.7 ± 5.4 bpm (*n* = 21) (Table 2). Median baseline VR (range) was 127.0 (79, 164) (*n* = 21). In the subset with AF‐RVR, the mean VR ± SEM prior to drug administration was 138.3 ± 4.3 bpm (*n* = 17). The effect of etripamil on VR at 1‐min intervals over a 60‐min period is shown (Figure 1). The average maximum reduction in VR ± SEM was 27.4 bpm ± 6.1 at 22 min. In total, 6 of 21 AF episodes (28.6%), converted to NSR within 60 min of etripamil (Figure 1 and Table 2); mean VR ± SEM reduction from baseline (bpm) up to 1 min prior to conversion was 37.5 ± 12.3. Mean and median reductions of VR from baseline at 15, 30, 45, and 60 min are reported in Table 2. Among 21 adjudicated AF episodes with ECG data 0–60 min post etripamil administration, individual heart rate nadir values ranged from 55 to 132 bpm. The mean individual nadir (minimum HR per episode) was 92 bpm.

**Table 2:** Effect of self‐administered etripamil on AF episode VR.

VR change from baseline following self‐administered etripamil					
	Baseline, 0 min	15 min	30 min	45 min	60 min
Adjudicated AF episodes with ECG data, *n* a	21	20	19	17	14^b^
Mean VR, bpm	129.7	104.6	110.7	103.9	110.2
SD	24.8	28.7	27.6	30	28.5
SEM	5.4	6.4	6.3	7.3	7.6
Median VR, bpm (range)	127	96	108	98	114
	(79, 164)	(67, 152)	(58, 154)	(63, 178)	(65, 161)
Mean change in VR from baseline, bpm		−23.2	−18.4	−22.4	−16.2
SD		25.6	28	27.1	21
SEM		5.7	6.4	6.6	5.6
Median change in VR from baseline, bpm (range)		−28.5	−22	−23	−18
		(−67.0, 36.0)	(−80.0, 52.0)	(−75.0, 33.0)	(−43.0, 34.0)

Abbreviations: AF, atrial fibrillation; BPM, beats per minute; ECG, electrocardiograph; Min, minutes SD, standard deviation; SEM, standard error of mean VR, ventricular rate.

^a^ ECG data for episodes were excluded after converting to NSR within 60 min (*n* = 6).

^b^ One patient had no ECG data available after 55 min

The safety analysis included all adjudicated AF episodes (26 episodes, Table 1). Self‐administered etripamil was well‐tolerated, with mild or moderate adverse event (AEs) symptoms occurring in > 1 episode, including rhinorrhea (*n* = 4), nasal discomfort (*n* = 4), and nasal congestion (*n* = 3). No serious AEs were observed, including 1 patient who self‐administered 2 doses separated by 22 min (Table 2). Bradyarrhythmias, AV block, or sinus pauses ≥ 3 s (either conversion pauses or pauses during AF), were not observed, including among patients taking concomitant medications (*n* = 11).

## 4 Discussion

In this open‐label study, etripamil was self‐administered outside of the healthcare setting without medical assistance. Unlike prior phase three studies, these patients had not received a test dose prior to their first use of etripamil. In patients with prior documented PSVT who experienced episodes of AF‐RVR, there was a rapid and substantial reduction in VR (maximal reduction in 22 min) with self‐administered etripamil, with an effect lasting at least 30–60 min. In the previously reported ReVeRA‐201 study of patients in AF‐RVR (randomized, double‐blind, placebo‐controlled study; drug administered by medical staff), the mean maximum reduction in VR over 60 min was 34.97 (95% CI, −45.13 to −24.81); placebo‐corrected: 29.91 bpm (95% CI, −40.31 to −19.52; *p* < 0.0001); and VR reductions were sustained for ≥ 150 min [15]. The authors speculate that early VR reduction and symptom relief following etripamil may attenuate the perpetuation of AF‐RVR and note the benefit of fast‐acting intranasally administered rate control before potential use of additional oral therapy. The sustained reduction in VR observed in the present analysis at later time points (30–60 min following drug administration), and the duration of reduction shown in ReVeRA‐201, indicates a pharmacodynamic effect of etripamil that lasts close to or longer than the drug's pharmacokinetic profile (half‐life of 2.5 h) [8, 15].

Etripamil effectively controlled rapid rates in AF‐RVR patients with and without concomitant medications. Prior use of AV nodal‐acting medication did not prevent RVR at the onset of paroxysmal AF. As previously noted, no patient on prior AV‐nodal blocking drugs had post‐treatment VR below 55 bpm or pauses greater than 3 s, further demonstrating the safety of etripamil. Higher doses of chronic β‐blockers or CCBs could exacerbate side effects such as hypotension, bradycardia, especially in patients with comorbidities. Additionally, oral medications are limited by slow absorption with time to peak effect taking hours, which often fails to provide immediate relief during symptomatic AF episodes, prolonging symptoms and potentially leading to complications.

Patients often seek urgent care for AF‐RVR episodes due to symptoms related to their rapid pulse. Promptly initiated rate‐control and symptom management could reduce the need for emergency room visits. Prompt rate‐ and symptom‐control likely attenuates a “snowball” effect whereby RVR begets RVR due to anxiety and higher adrenergic tone. Current treatment options, such as orally administered β‐blockers and CCBs, have slow onsets of action and potential side effects, limiting their efficacy to quickly reduce rapid rates and symptoms. Rapidly deployed intranasal therapy might result in faster and more complete symptom relief and potentially permit a “wait and see” approach for patients, thus preventing ED visits and reducing healthcare resource utilization. Additional investigation of patient‐reported outcomes (e.g., symptom relief) to demonstrate the potential improvement of AF‐RVR symptoms following self‐administration of etripamil would be valuable.

### 4.1 Study Limitations

The study was not designed to analyze the efficacy or safety of etripamil in patients with AF. Patients had prior PSVT with or without prior AF and may not represent a typical AF population. Etripamil administration may have occurred prior to or just after the start of ECG recording, at a variable time after symptom onset which could have affected the baseline VR measure.

## 5 Conclusions

In this post hoc analysis of the NODE‐303 study, self‐administered etripamil was observed to reduce the rapid ventricular rates of patients with symptomatic AF. These findings warrant further studies on the efficacy and safety of etripamil to acutely control symptomatic AF‐RVR.

## Funding

The authors received no specific funding for this work.

## Conflicts of Interest

P.D., J.E.I., J.P.P., and A.J.C. are consultants for Milestone Pharmaceuticals. M.A., B.S.S., P.D., and A.J.C. serve on the steering committee for Milestone Pharmaceuticals. J.P.P. has received grants for clinical research from Abbott, the American Heart Association, the Association for the Advancement of Medical Instrumentation, Bayer, Boston Scientific, iRhythm, and Philips and serves as a consultant to Abbott, AbbVie, ARCA biopharma, Bayer, Boston Scientific, Bristol Myers Squibb (Myokardia), Element Science, Itamar Medical, LivaNova, Medtronic, ElectroPhysiology Frontiers, ReCor, Sanofi, Philips, and UpToDate. S.S., M.S., and D.B.B. are employees and shareholders of Milestone Pharmaceuticals. A.J.C. has received grants and personal fees from Boehringer Ingelheim, Bayer, Pfizer, Bristol‐Myers Squibb, and Daiichi Sankyo; personal fees from Medtronic, Boston Scientific, Menarini, and Biotronik; and support from Anthos, Sanofi, Abbott, GlaxoSmithKline, and Johnson & Johnson.
